# Retinoblastoma gene expression profiling based on bioinformatics analysis

**DOI:** 10.1186/s12920-023-01537-4

**Published:** 2023-05-13

**Authors:** Jun Mao, Mingzhi Lu, Siduo Lu, Yiqiao Xing, Xuejiao Xu, Ying Chen, Huirong Xu, Wei Zuo, Jingwen Zhou, Wei Du

**Affiliations:** 1 The Eighth Affiliated Hospital of Sun Yat-sen University, Shenzheng, China; 2 Aier Eye Hospital of Wuhan University, Wuhan, China; 3 The First Affiliated Hospital of Kunming Medical University, Yunnan, China

**Keywords:** Retinoblastoma, Bioinformatics analysis, Biomarkers, ceRNA, Regulatory network

## Abstract

**Background:**

Retinoblastoma (RB) is frequently occurring malignant tumors that originate in the retina, and their exact cause and development mechanisms are yet to be fully comprehended. In this study, we identified possible biomarkers for RB and delved into the molecular mechanics linked with such markers.

**Methods:**

In this study GSE110811 and GSE24673 were analyzed. Weighted gene co-expression network analysis (WGCNA) was applied to screen modules and genes associated with RB. By overlapping RB-related module genes with differentially expressed genes (DEGs) between RB and control samples, differentially expressed retinoblastoma genes (DERBGs) were acquired. A gene ontology (GO) enrichment analysis and a kyoto encyclopedia of genes and genomes (KEGG) enrichment analysis were conducted to explore the functions of these DERBGs. To study the protein interactions of DERBGs, a protein–protein interaction (PPI) network was constructed. Hub DERBGs were screened using the least absolute shrinkage and selection operator (LASSO) regression analysis, as well as the random forest (RF) algorithm. Additionally, the diagnostic performance of RF and LASSO methods was evaluated using receiver operating characteristic (ROC) curves and single-gene gene set enrichment analysis (GSEA) was conducted to explore the potential molecular mechanisms involved with these Hub DERBGs. In addition, the competing endogenous RNA (ceRNA) regulatory network of Hub DERBGs was constructed.

**Result:**

About 133 DERBGs were found to be associated with RB. GO and KEGG enrichment analyses revealed that the important pathways of these DERBGs. Furthermore, the PPI network revealed 82 DERBGs interacting with each other. By RF and LASSO methods, *PDE8B*, *ESRRB*, and *SPRY2* were identified as Hub DERBGs in patients with RB. From the expression assessment of Hub DERBGs, it was found that the levels of expression of *PDE8B*, *ESRRB*, and *SPRY2* were significantly decreased in the tissues of RB tumors. Secondly, single-gene GSEA revealed a connection between these 3 Hub DERBGs and oocyte meiosis, cell cycle, and spliceosome. Finally, the ceRNA regulatory network revealed that hsa-miR-342-3p, hsa-miR-146b-5p, hsa-miR-665, and hsa-miR-188-5p may play a central role in the disease.

**Conclusion:**

Hub DERBGs may provide new insight into RB diagnosis and treatment based on the understanding of disease pathogenesis.

**Supplementary Information:**

The online version contains supplementary material available at 10.1186/s12920-023-01537-4.

## Background

Retinoblastoma (RB), An ophthalmological common intraocular cancer, poses a serious threat to the vision and well-being of patients [1, 2]

Being a genetic disease, RB is caused by the deletion of the tumor suppressor gene, *BR1* [3]. With the advancement in gene detection technology, abnormal expression of genes such as *MYCN* has become an important factor in the progression of RB [4]. Due to the advancements in methods and innovation, RB treatment has undergone a noteworthy transformation from excision, radiotherapy, and intravenous chemotherapy to intra-arterial chemotherapy combined with local therapy [5–7].

While the pathogenesis of RB is unclear, the rate of eyeball removal in patients with advanced RB remains high, indicating the need for further treatment innovations. It is imperative to study the biological process (BP) and related potential mechanisms of RB to develop a new treatment strategy. Researchers have discussed the most important prognostic factors and potential mechanisms of RB through the use of existing data on RB and bioinformatics methods [8, 9]. For example, an analysis conducted by Wen et al. [10] identified two critical microRNA targets in RB: let-7a and let-7b by analyzing a variety of bioinformatics studies and identifying microRNA-target gene-transcription factor regulatory networks in RB. According to Gao et al. [11] the long noncoding RNA (lncRNA) MEG3 may play a role in tumor suppression in RB, and the activation of Lnc00152 by Sp1 induces EMT through the miR-30d/SOX9/ZEB2 pathway and enhances the invasion and metastasis of RB cells through this pathway. The pathogenesis of cancer is extremely complex, but more research needs to be conducted on this topic.

A better comprehension of the genetic, environmental, as well as immune-regulatory factors of RB may provide important insights into its diagnosis and pathogenesis. Bioinformatics assessments have been used to diagnose many diseases, but enough assessments in RB have not been performed. This study aims to identify the biomarkers of RB prognosis using multiple bioinformatics-based datasets and explain its pathogenesis using functional enrichment analysis and the competing endogenous RNA (ceRNA) network. These findings may contribute to an additional understanding of the pathogenesis of RB and guide future research on this disease.

## Materials and methods

### Source of data

A total of two RB datasets, GSE110811 and GSE24673, were retrieved out of the Gene Expression Omnibus (GEO) (https://www.ncbi.nlm.nih.gov/geo/). GSE110811 (19 retinal tissue samples and 31 RB samples) and GSE24673 (2 cadaveric eye samples and 9 RB samples) were subsequently merged into a new dataset that contained 40 RB samples and 21 normal samples.

### Weighted gene co-expression network analysis (WGCNA)

The gene-expression profiles from the GSE110811 and GSE24673 datasets were used to investigate the RB-associated module via the “WGCNA” R package (version 1.70-3) [12]. Initially, an adjacency matrix was constructed based on the formula for the adjacency matrix to describe the correlation strength between the nodes [13].

$$s_{ij} = \left| {cor\left( {x_{i} ,x_{j} } \right)} \right|a_{ij} = s_{ij} \beta$$

where, *i* and *j* represent two different genes, and *x*_i_ and *x*_j_ represent expression scores of the corresponding genes. *S*_ij_ represents the correlation coefficient, and *a*_ij_ represents the strength of the correlation between *i* and *j*. In this research, the adjacency matrix was constructed with a scale-free topological index of 0.85 and an optimal soft-threshold power ($\beta$) of 12. The adjacency matrix was then converted into a matrix of topological overlap. Finally, hierarchical clustering trees were constructed by dynamically cutting trees (module sizes of 25) for identifying key modules through the introduction of genes with similar expression patterns into the same module.

### Differential genes expression analysis

As a first step, differentially expressed genes (DEGs) between RB samples and normal samples in the merged dataset were identified using the “limma” R package (version 3.46.0), with an adjusted *p* value of < 0.05 and a |log2FC|> 1 [14]. To obtain differentially expressed retinoblastoma genes (DERBGs), the DEGs were intersected with key module genes using the VennDiagram R package (version 1.6.20) [15]. To evaluate the potential functions of DERBGs, a Gene Ontology (GO) function and the Kyoto Encyclopedia of Genes and Genomes (KEGG) pathway enrichment analyses were conducted using the clusterProfiler R package [16–20].

### Protein–protein interaction (PPI) network construction

To investigate if there are protein interactions between DERBGs, the Search tool to retrieve Interacting Genes and Proteins (STRING) website (https://string-db.org) was used to map a PPI network of these DERBGs. Further, the PPI network was visualized using Cytoscape, and the top ten DERBGs were identified using the maximal clique centrality (MCC) algorithm of Cytohubba [21].

### Screening and validation of Hub DERBGs

Hub DERBGs were screened using the random forest (RF) method with the “Randomforest” R package (version 4.7-1) and the least absolute shrinkage and selection operator (LASSO) regression assessment with the “glment” R package (version 4.1-1) [21, 22]. Besides, the “pROC” R package (V 1.17.0.1) was used to evaluate the diagnostic performance of the RF and LASSO methods [23]. Following this, the Hub DERBG expression values were validated in the merged dataset (*p* < 0.05).

### Single-gene gene set enrichment analysis (GSEA)

To explore the regulatory pathways and biological functions associated with these Hub DERBGs, the “clusterProfiler” R package (version 3.18.0) was used to perform the GSEA of each DERBG [16, 17]. An adjusted *p* value of < 0.05 was used to indicate significant thresholds for GSEA.

### Construction of a ceRNA regulatory network

Differentially expressed microRNAs (DEmiRNAs) were identified in the GSE41321 dataset (*p* < 0.05 and |logFC|> 1) using the “limma” R package (version 3.46.0) [14]. Meanwhile, the miRWalk DB (http://mirwalk.umm.uni-heidelberg.de/) was utilized for predicting miRNAs from the Hub DERBGs (binding *p* > 0.95 and energy < − 15). To obtain the miRNA–messenger RNA (mRNA) relationship pair and common miRNAs, the predicted miRNAs were intersected with DEmiRNAs. Further, the “DEseq” R package (V1.34.0) was used to identify differentially expressed long noncoding RNAs (DElncRNAs) in the GSE125903 dataset (*p* < 0.05 and |logFC|> 1) [24]. Also, based on the common miRNAs, the Starbase database (http://starbase.sysu.edu.cn/) was employed to predict lncRNAs (binding *p* > 0.95 and energy < − 15) [25]. Finally, a ceRNA regulatory network was constructed by integrating the interactions between DEmiRNAs and DElncRNAs. To visualize the ceRNA network, the Cytoscape R package (V3.8.2) was used [21].

### Statistical analysis

R software (https://www.r-project.org/, V4.0.3) was used to perform statistical analyses and to visually plot the results. Using Pearson correlation analysis, correlation coefficients and *p* values were calculated for the RB-related module and patients with RB. A *p* value of < 0.05 was considered statistically significant.

## Results

### Identification of the RB-associated modules and genes through WGCNA

**Fig. 1:**
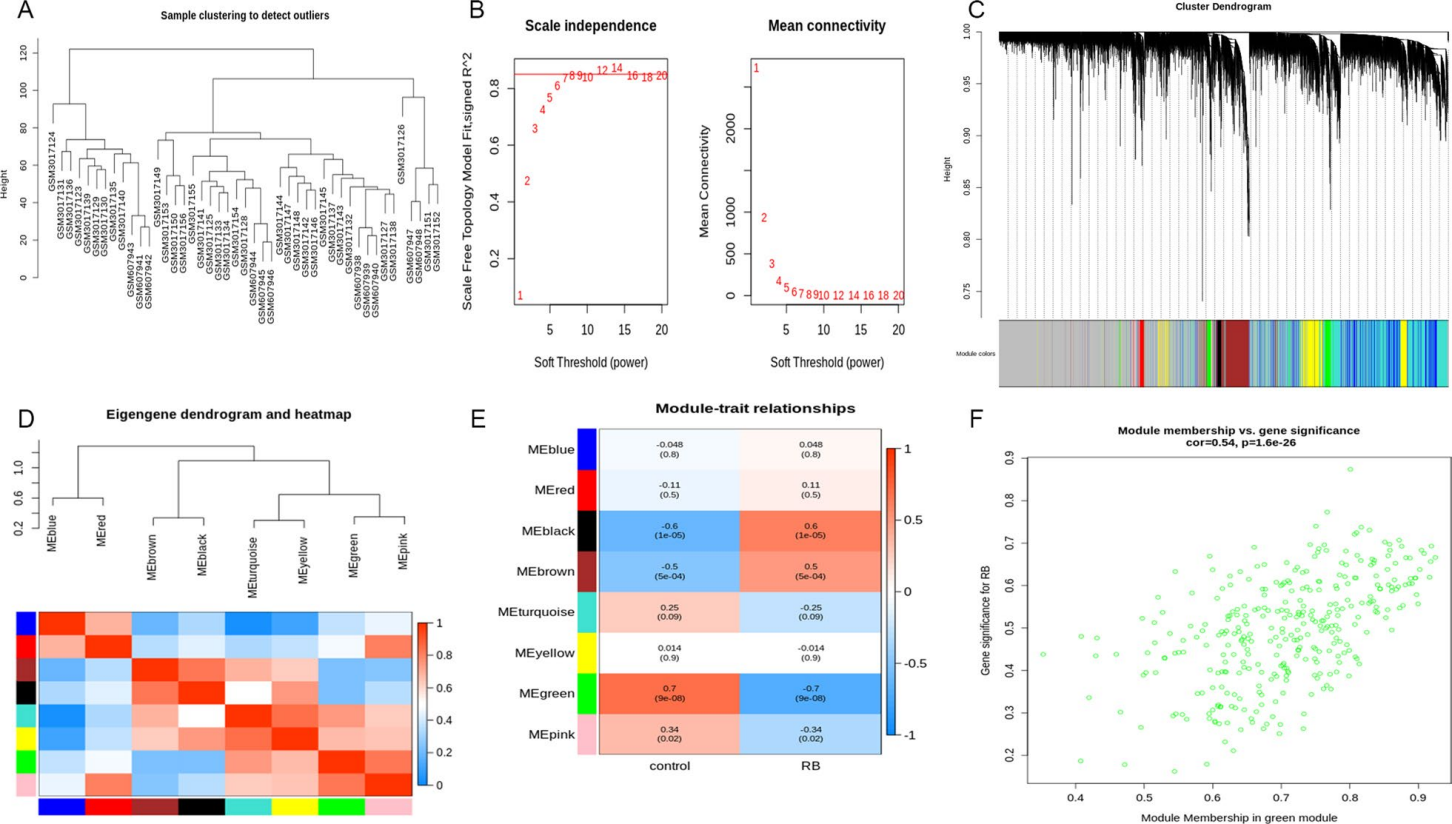
Weighted gene co-expression network analysis results. **A** Sample dendrogram and trait heatmap; **B** Scale independence and mean connectivity; **C** Cluster dendrogram; **D** Eigengene dendrogram and eigengene heatmap; **E** Module trait relationships; **F** Scatter plot of genes in the green module

A co-expression network was constructed by applying WGCNA to all samples and genes in the merged dataset to identify RB-related modules and genes. Additional file 1 provides a comprehensive overview of the Principal Component Analysis (PCA) carried out on the gene expression datasets. The sample dendrogram in Fig. 1A indicates that the merged dataset does not contain any outliers. Following this, a scaleless network was constructed with a 12 soft-threshold power (β), and an index of scale-free topologies of 0.85 was set (Fig. 1B). Using a dynamic tree-cutting method to introduce genes with similar expression patterns into a single module (module size = 25), a hierarchical clustering tree with 8 modules was constructed (Figs. 1C and D). As shown in Fig. 1E and F, MEgreen has the highest correlation with RB (*Cor* = 0.54, *p* = 1.6e−26). Therefore, a total of 332 genes from the MEgreen module were analyzed in the following steps.

### Identification of DERBGs in RB

**Fig. 2:**
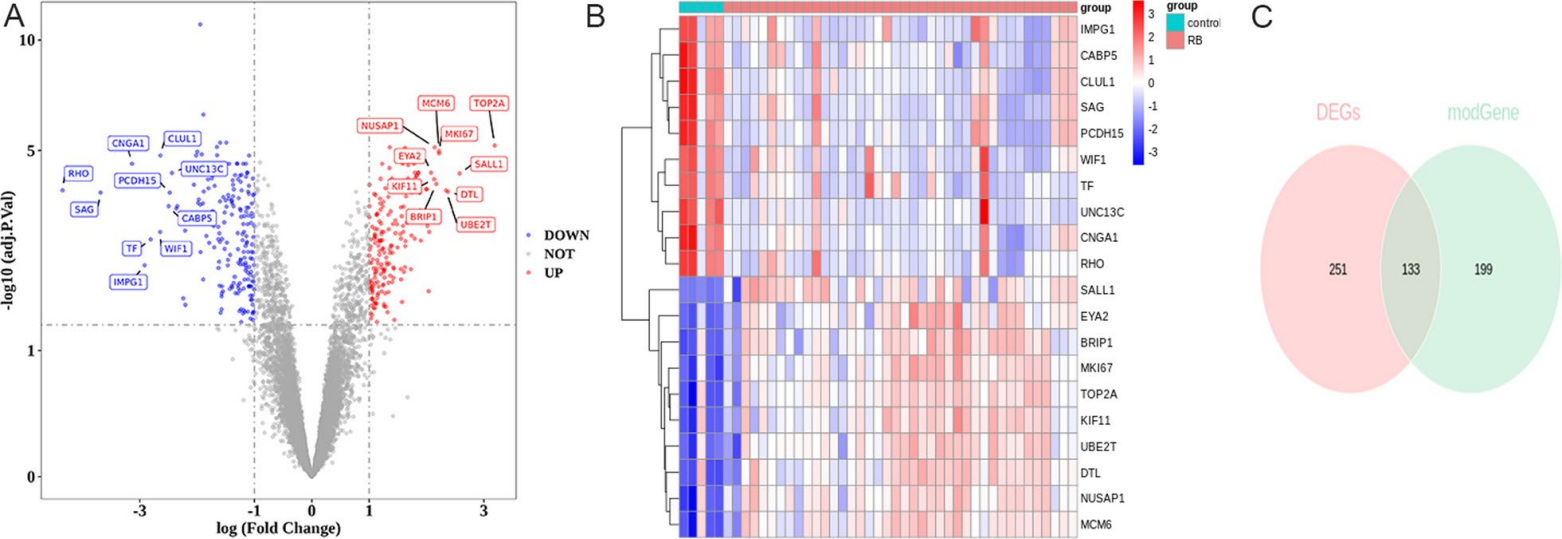
Genome-wide analysis of gene expression of retinoblastoma (RB). **A** A volcano plot of differentially expressed genes (DEGs); **B** Heatmap of DEGs; **C** Venn diagram of DEGs detected among RB DEGs and RB module genes

The first step in identifying DERBGs associated with RB was to screen DEGs between RB samples and normal samples in the merged dataset. As illustrated in Fig. 2A and B, a total of 384 DEGs were identified in RB samples, of which 188 were downregulated and 196 were upregulated. Following this, 133 DERBGs were obtained for further analysis by intersecting DEGs with genes related to RB modules (Fig. 2C).

### PPI network of DERBGs and functional analysis

**Fig. 3:**
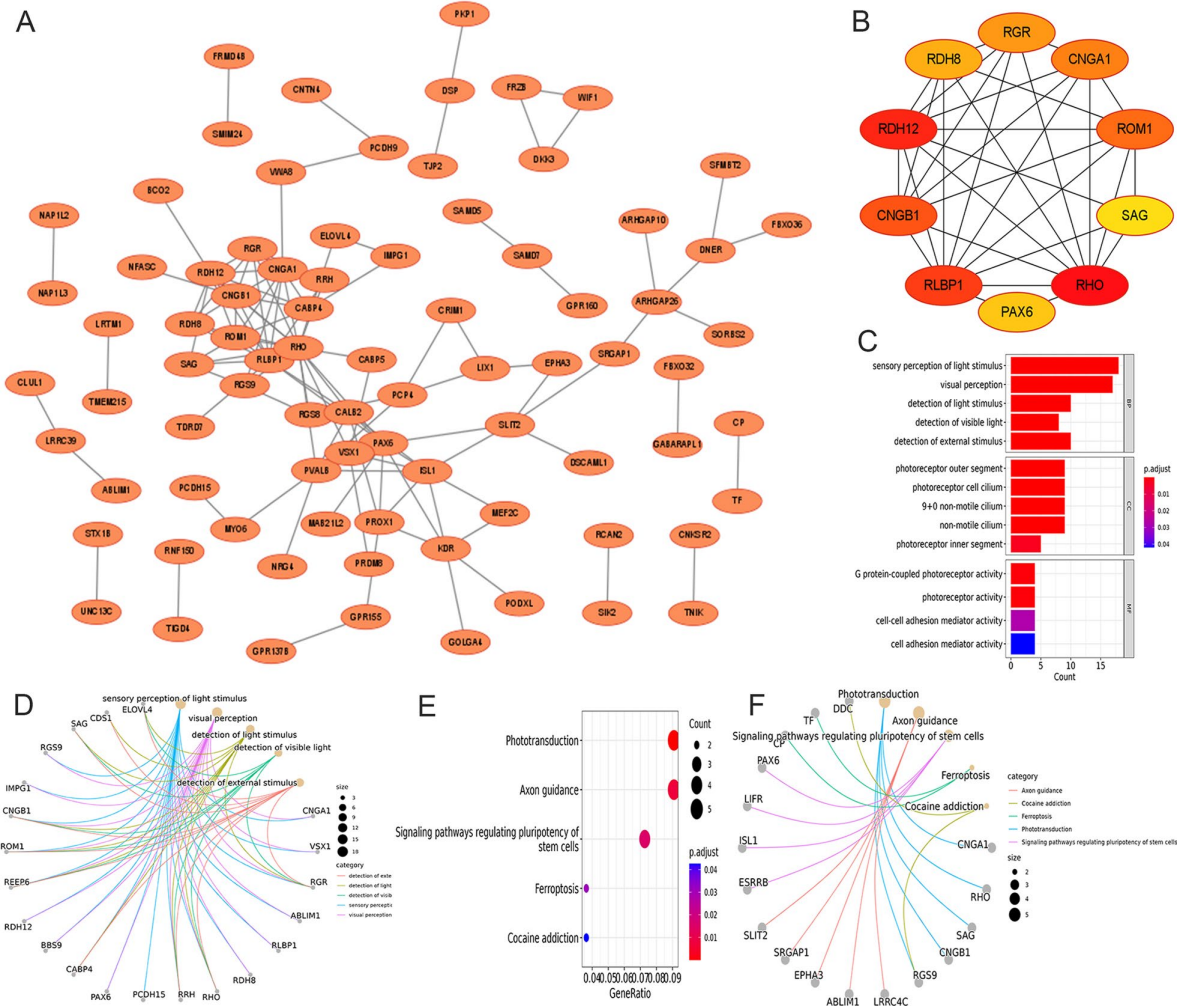
A protein–protein interaction (PPI) network of differentially expressed retinoblastoma genes (DERBGs) and functional analysis. **A** PPI network; **B** The top 10 genes; **C** Gene Ontology (GO) enrichment of DERBGs; **D** Chord plot of GO enrichment; **E** Kyoto Encyclopedia of Genes and Genomes (KEGG) pathway enrichment of DERBGs; **F** Chord plot of the KEGG pathway enrichment

A network map of the PPI network was developed using the STRING website to study the interactions among 133 DERBGs. It was possible to obtain a PPI network with 118 interactions and 82 nodes (Fig. 3A). To rank these DERBGs with interactions, the MCC algorithm of the Cytoscape software was used (Fig. 3B; *RDH8*, *RGR*, *CNGA1*, *ROM1*, *SAG*, *RHO*, *PAX6*, *RLBP1*, *CNGB1*, and *RDH12*). To explore the role of 82 DERBGs in BPs, GO and KEGG were constructed. The clustering coefficient was 0.267. As a result of GO enrichment analysis, these DERBGs were primarily involved in five terms. For example, in BPs, the light was perceived as a sensory stimulus, visual stimulus, and detectable stimulus; in addition, these DERBGs were primarily involved in five terms, such as photoreceptor outer segment, photoreceptor cell cilium, and 9 + 0 non-motile cilium in the cellular component (CC); in molecular function (MF), these DERBGs were mainly engaged in G-protein-coupled photoreceptor activity, photoreceptor activity, the activity of cell–cell adhesion mediators, and the activity of cell adhesion mediators (Fig. 3C and D). According to the KEGG pathways, these DERBGs are significantly associated with phototransduction, axon guidance, pathways of signal transmission regulating pluripotency, ferroptosis, and cocaine addiction pathways (Fig. 3E and F).

### Screening and expression level validation of Hub DERBGs

**Fig. 4:**
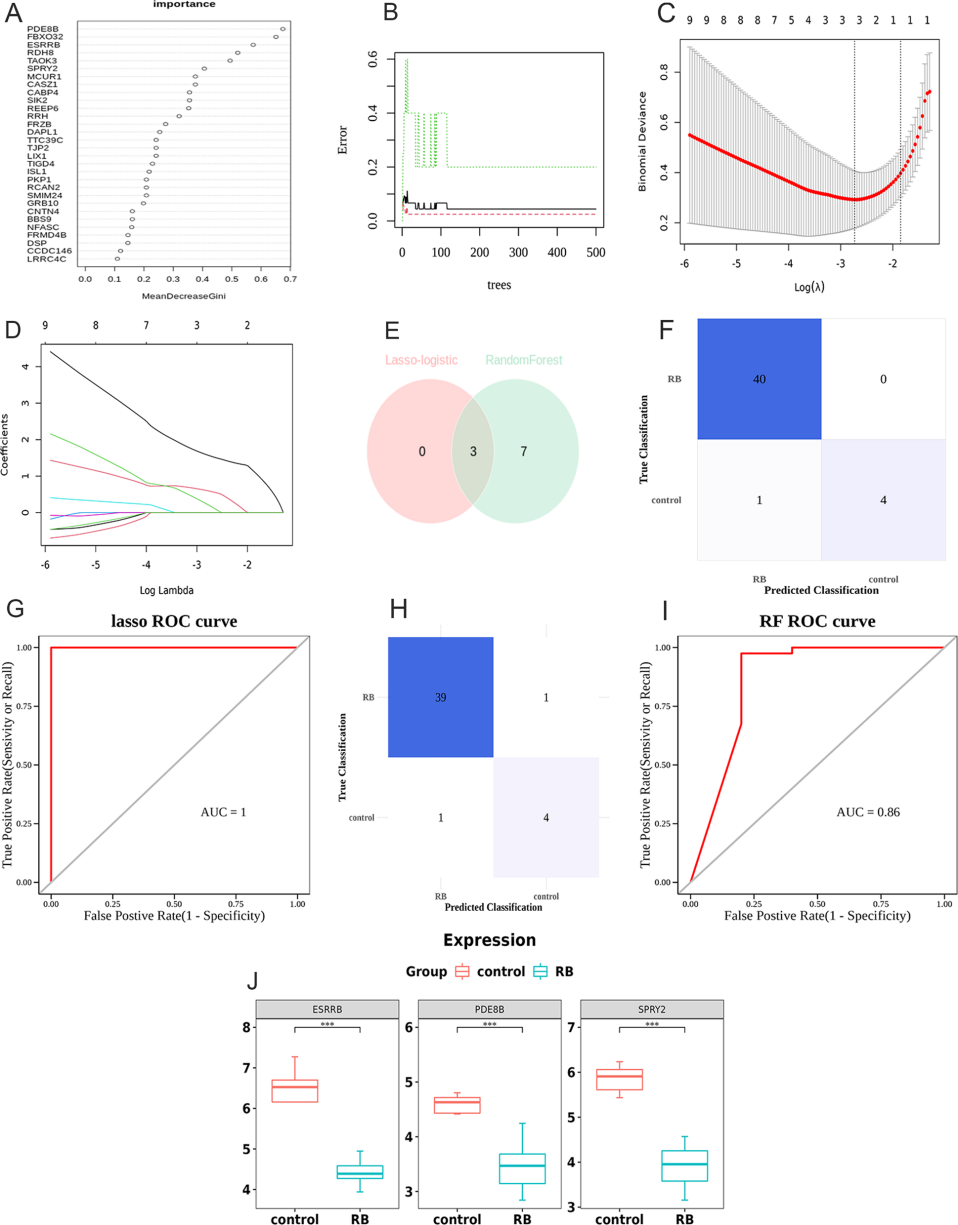
Screening and expression level validation of Hub differentially expressed retinoblastoma genes (DERBGs). **A** Importance ranking of the top 10 DERBGs; **B** Random forest algorithm; **C** Lasso-logistic algorithm; **D** Lasso result graph; **E** Venn diagram of differentially expressed genes (DEGs) detected using the random forest algorithm and Lasso-logistic algorithm; **F** Confusion matrix heat map of the Lasso-logistic algorithm; **G** Receiver operating characteristic (ROC) curve analysis of the Lasso-logistic algorithm; **H** Confusion matrix heat map of the random forest algorithm; **I** ROC curve analysis of the random forest algorithm; **J** messenger RNA (mRNA) expression levels of PDE8B, ESRRB, and SPRY2

The RF algorithm was used to identify the top ten DERBGs (*PDE8B, FBXO32, ESRRB, RDH8, TAOK3, SPRY2, MCUR1, CASZ1, CABP4,* and *SIK2*) from the merged dataset for further validation and selection of Hub DERBGs with significantly characteristic value for classifying RB and normal samples (Fig. 4A and B). Additionally, three DERBGs were selected using the LASSO algorithm (lambda min = 0.06493903) (Figs. 4C and D). By integrating the DERBGs screened by the RF and LASSO algorithms, a total of 7 DERBGs were identified, of which 3 (*PDE8B*, *ESRRB*, and *SPRY2*) were selected simultaneously by both algorithms (Fig. 4E). For classification and diagnostic purposes, those DERBGs were identified as Hun DERBGs. Further, the receiver operating characteristic (ROC) curve analysis and the confusion matrix heat map together demonstrate that RF and LASSO algorithms can provide good diagnostic performance (Figs. 4F–I). The merged dataset was used to validate the levels of expression of three Hub DERBGs (*PDE8B*, *ESRRB*, and *SPRY2*). In RB tumor tissue, the levels of mRNA of *PDE8B*, *ESRRB*, and *SPRY2* were greatly reduced (Fig. 4J).

### Singe-gene GSEA of Hub DERBGs in RB

**Fig. 5:**
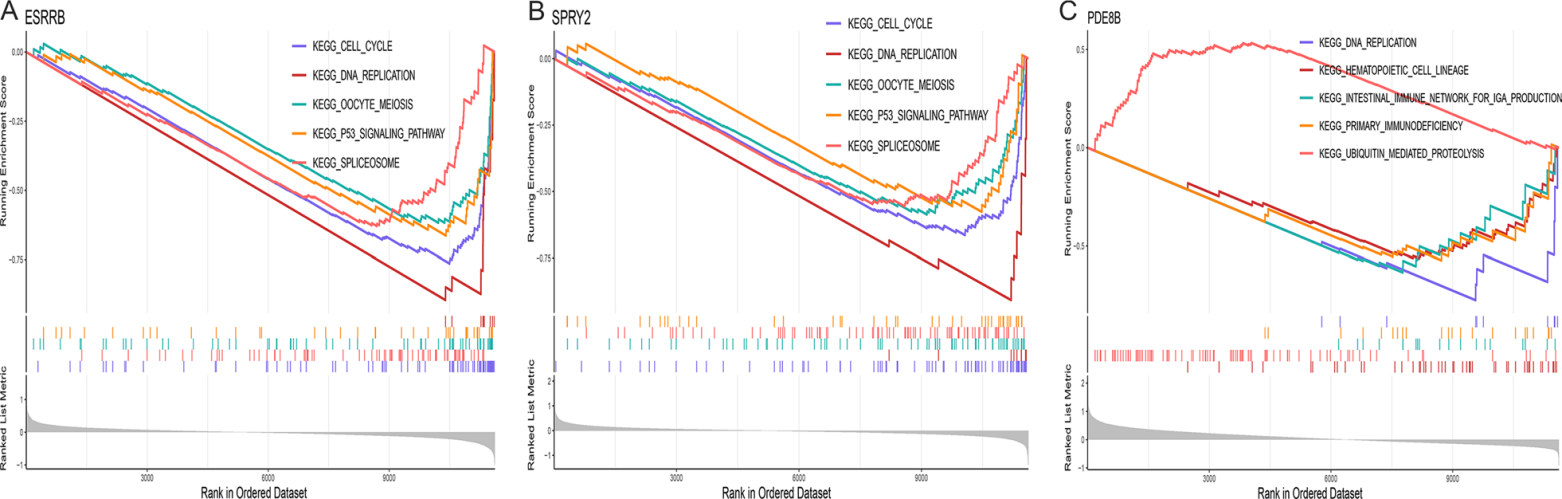
Significantly enriched pathways of Hub differentially expressed retinoblastoma genes in retinoblastoma obtained by gene set enrichment analysis. **A** Enrichment plots for the five key pathways abnormally activated in estrogen-related receptor beta; **B** Enrichment plots for the five key pathways abnormally activated in Sprouty RTK signaling antagonist 2; **C** Enrichment plots for the five key pathways abnormally activated in phosphodiesterase 8B

A single-gene GSEA based on the KEGG gene sets was performed to determine the molecular mechanisms involved in Hub DERBGs in RB. As shown in Fig. 5, the top five KEGG pathways enriched by each Hub DERBG were identified. The estrogen-related receptor beta* (ESRRB)* gene was associated with cell cycle, spliceosome, and oocyte meiosis, as well as the p53 signaling pathway and DNA replication (Fig. 5A). Figure 5B shows the association between *SPRY2* and cell cycle, spliceosome, DNA replication, p53 signaling pathway, and meiosis of oocytes. There was a correlation between *PDE8B* and DNA replication, hematopoietic cell lineage, intestinal immune network for IgA production, primary immunodeficiency, and ubiquitin-mediated proteolysis (Fig. 5C).

### Construction and assessment of a ceRNA regulatory network for Hub DERBGs in RB

**Fig. 6:**
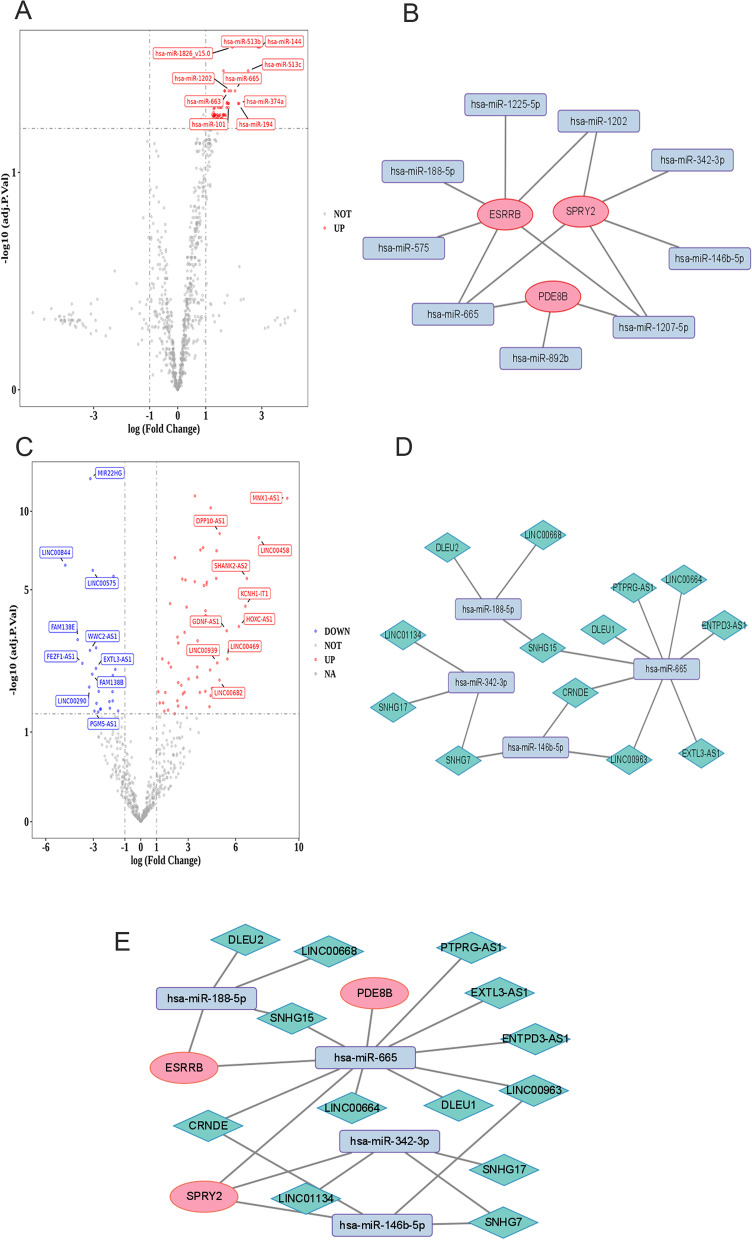
ceRNA regulatory network of Hub differentially expressed retinoblastoma genes in retinoblastoma. **A** Volcano plot of differentially expressed miRNAs (DEmiRNAs) in the GSE41321 dataset; **B** DEmiRNAs network; **C** Volcano plot of differentially expressed long noncoding RNAs (DElncRNAs) in the GSE125903 dataset; **D** mi-LncRNAs network; **E** ceRNA network

To identify additional miRNAs and lncRNAs that may regulate the expression of Hub DERBGs, 54 DEmiRNAs were detected in the GSE41321 dataset, all of which were upregulated (Fig. 6A). Meanwhile, the miRWalk database was applied to predict miRNAs, and the predicted miRNAs were intersected with DEmiRNAs to determine the nine common miRNAs (hsa-miR-1225-5p, hsa-miR-1202, hsa-miR-342-3p, hsa-miR-146b-5p, hsa-miR-1207-5p, hsa-miR-892b, hsa-miR-665, hsa-miR-575, and hsa-miR-188-5p; Fig. 6B). Moreover, 83 DElncRNAs in the GSE125903 dataset were identified, of which 59 DElncRNAs were upregulated and 24 DElncRNAs were downregulated (Fig. 6C). In addition, based on these 9 common miRNAs, 13 lncRNAs (DLEU2, LINC00668, SNHG15, CRNDE, DLEU1, PTPRG-AS1, LINC00664, ENTPD3-AS1, EXTL3-AS1, LINC00963, SNHG7, SNHG17, and LINC01134) and 4 miRNAs (hsa-miR-342-3p, hsa-miR-665, hsa-miR-185) were analyzed using the Starbase database (Fig. 6D). Finally, interactions between these miRNAs and lncRNAs were integrated to construct a ceRNA regulatory network, and the visualization of the network was carried out using Cytoscape (Fig. 6E).

## Discussion

RB is one of the most serious eye diseases that can result in blindness, disability, and even death in infants [26–28]. Thus, the progression of novel strategies to diagnose and treat RB requires a detailed understanding of the mechanisms involved.

Additional file 2 illustrates the flow chart of the present study. This research aimed to identify RB DEGs and RB-associated gene modules using a systems biology approach called WGCNA. About 384 DEGs and nine module-clinical trait relationships significantly correlated with RB, which suggests that these module genes contribute significantly to the occurrence and progression of RB. From cross-DEG and RB-associated module genes, 133 DERBGs were obtained for further analysis. Further, functions and pathways involved in RB pathogenesis were examined. Fourteen GO terms and 5 KEGG pathways that were significantly enriched were identified. Among them, the notable ones are the pathways regulating the pluripotency of stem cells and ferroptosis. Certain cancers involve pathways of signal transmission that regulate stem cell pluripotency [29]. A new type of cell death characterized by distinct properties and recognizing functions that may be associated with physical conditions or different diseases, such as cancer, is called ferroptosis [30].

PPI networks of DEGs were built. *RDH8, RGR, CNGA1, ROM1, SAG, RHO, PAX6, RLBP1, CNGB1*, and *RDH12* were identified as the ten most important Hub genes identified from PPI analysis. A previous bioinformatics study on RB also showed that *ROM1*, *CNGB1*, and *RDH12* may have a role in predicting the progress of RB, which is consistent with the findings of this research [31]. Previous studies have shown that *SAG* is a prospective target that could further be explored as a potential candidate in therapy and may further assist in understanding the mechanism of RB [32]. SAG is related to photoreceptors, which are the “cell of origin” in RB. The proteins might likely participate in unidentified pathways in RB. Interestingly, *RGR*, *CNGA1*, and *RLBP1* play an important role in retinitis pigmentosa. Whether they also play an important role in RB needs further investigation [33]. It can be inferred that the progression of RB may be significantly influenced by these genes.

The RF and Lasso logistics diagnostic models revealed the top three genes with the highest score degree, namely *PDE8B, ESRRB,* and *SPRY2*. *PDE8B* (phosphodiesterase 8B) is a gene encoding an enzyme that catalyzes the hydrolysis of a secondary messenger molecule, cAMP, by cyclic nucleotide phosphodiesterase (PDE). In addition, *SPRY2* (Sprouty RTK Signaling Antagonist 2) encodes a protein that belongs to the Sprouty family [34–37]. Outcomes from GSEA were enriched for p53 SIGNALING PATHWAY and SPLICEOSOME, in agreement with previous findings, indicating that the gene plays an important role in RB initiation and development [38, 39].

In an extensive range of processes, *ESRRB*, a protein-coding gene, plays an important role in the cell cycle, spliceosomes, and oocyte meiosis, as well as the p53 signaling pathway and DNA replication [40, 41]. Among them, HEMATOPOIETIC CELL LINEAGE, PRIMARY IMMUNODEFICIENCY, and UBIQUITIN-MEDIATED PROTEOLYSIS are strongly associated with the mechanism of cancer [42–45]. Nevertheless, the role of ESRRB in the progression of RB remains unknown.

Competitive endogenous RNA networks elucidate the mechanisms of RNA interactions that serve as key players in numerous biological processes. Although the precise mechanisms are yet to be fully understood, it is evident that these noncoding RNAs assume distinct functions in RB development. For example, study has shown that part of lncRNA DANCR can increase tumor aggressiveness [46]. The other study also showed that the lncRNA UCA1 promotes carboplatin resistance in RB cells by acting as a ceRNA for miR-206 [47]. Our study has revealed novel regulatory networks that were not previously reported. The regulatory analysis of the ceRNA network presents an opportunity for further validation and investigation through gene overexpression and knockout, as well as the use of double-luciferase reporter analysis.

The following are some of the highlights of this study. First, multiple datasets were used in this study, which resulted in stronger outcomes than those observed in previous studies. Second, two diagnostic models have been developed in this study for the first time before the selection of final key genes by crossover. From a comparative analytical perspective, this is worthy of further investigation. In conclusion, the follow-up analysis in this study was complete, and the constructed ceRNA could be used as a basis for further research. Nevertheless, a limitation of this study is that it was not possible to validate the results by Quantitative Real Time Polymerase Chain Reaction (qRT-PCR) due to the lack of clinical samples.

## Conclusions

To summarize, this study identified key genetic components and the functional pathways that may contribute to the progression of RB. In this study, Hub genes and pathways were identified that may contribute to a better understanding of the mechanisms underlying RB pathogenesis. Bioinformatics methods were used to construct a regulatory network for ceRNA related to RB. As well as identifying potential prognostic biomarkers, a deeper understanding of the development of RB tumors has been achieved. In the future, more experimental studies are required to validate the underlying biological regulatory mechanisms involved.

## Supplementary Information

**Additional file 1.** Principal Component Analysis (PCA) of Gene Expression Datasets.

**Additional file 2.** Flow Chart of the Bioinformatics Analysis.

## Data Availability

The datasets analyzed during the current study are available in the “GSE110811 and GSE24673”, (http://www.ncbi.nlm.nih.gov/geo/).

## Abbreviations

RB: Retinoblastoma
BP: Biological process
CC: Cellular component
MF: Molecular function
ceRNA: Competing endogenous RNA
GEO: Gene expression omnibus
DEGs: Differentially expressed genes
DERBGs: Differentially expressed retinoblastoma genes
KEGG: Kyoto encyclopedia of genes and genomes
PPI: Protein–protein interaction
STRING: Search tool to retrieve interacting genes and proteins
MCC: Maximal clique centrality
RF: Random forest
LASSO: Least absolute shrinkage and selection operator
DEmiRNAs: Differentially expressed micro RNAs
DElncRNAs: Differentially expressed long noncoding RNAs
PCA: Principal component analysis
qRT-PCR: Quantitative real time polymerase chain reaction
ROC: Receiver operating characteristic
