# Supplementary figures and images for: Retinoblastoma gene expression profiling based on bioinformatics analysis
Source: BMC Med Genomics. 2023 May 13;16:101. doi: 10.1186/s12920-023-01537-4 (PMC10183129; doi:10.1186/s12920-023-01537-4)

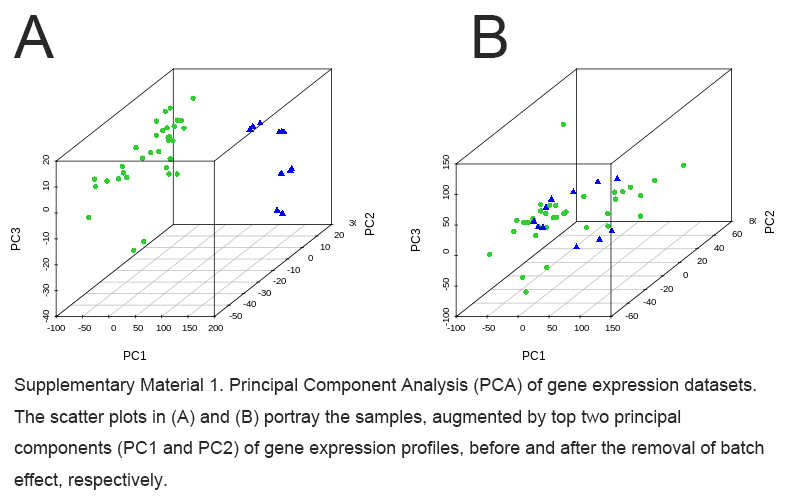

Supplement: Supplementary file 1 — Additional file 1. Principal Component Analysis (PCA) of Gene Expression Datasets. [file 12920_2023_1537_MOESM1_ESM.tif]

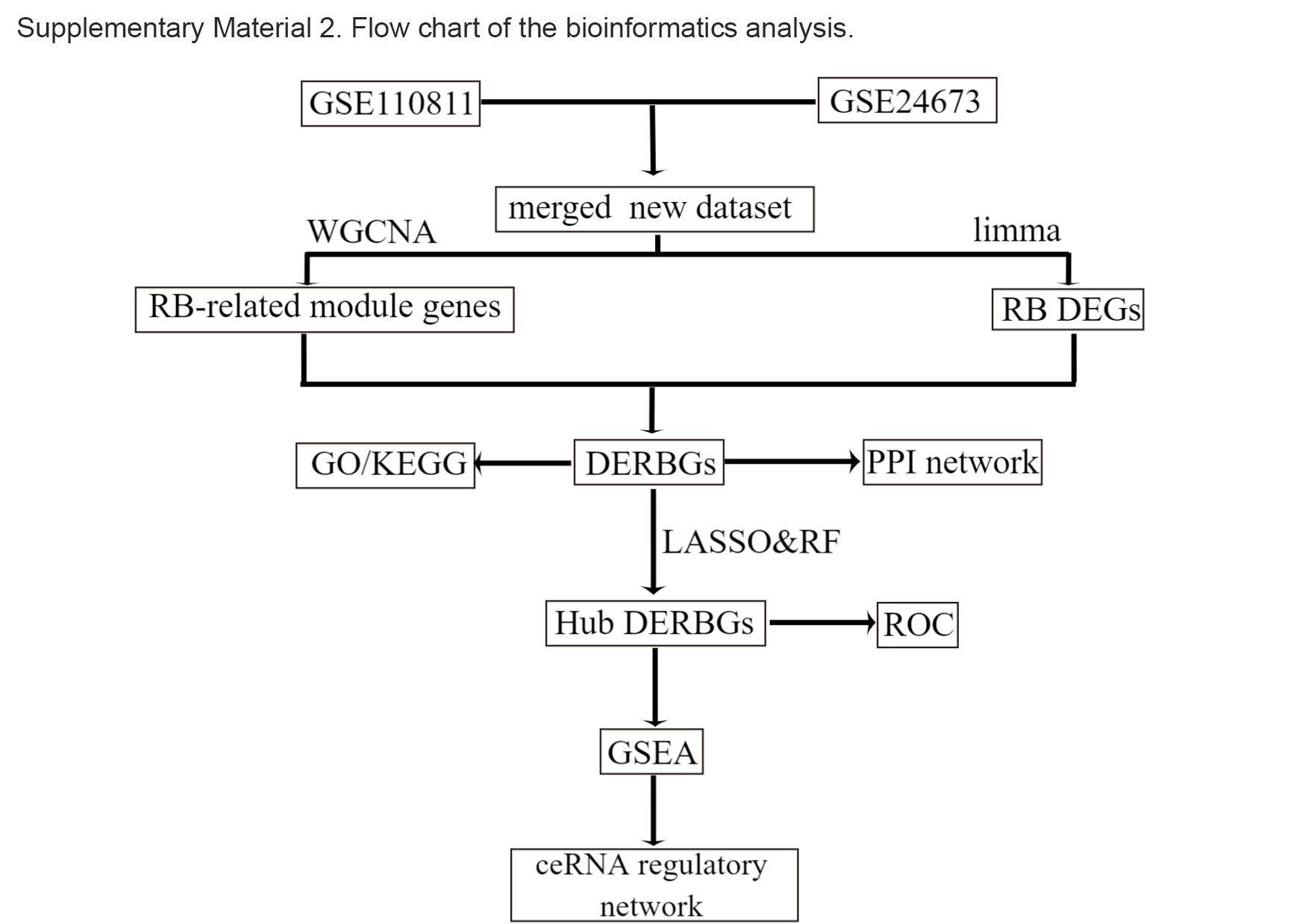

Supplement: Supplementary file 2 — Additional file 2. Flow Chart of the Bioinformatics Analysis. [file 12920_2023_1537_MOESM2_ESM.tif]
